# Population reach, feasibility and acceptability of digital therapeutics for smoking cessation among people living with HIV: Results of the Quitting Matters pilot trial

**DOI:** 10.1016/j.drugalcdep.2025.113015

**Published:** 2025-12-25

**Authors:** R. Vilardaga, F.J. McClernon, O. Akingbule, P. Mannelli, S.M. Thomas, J.M. Davis, M.F. Gray, C. Arnold, I. Chow Kai Reyes, R. Ashare, M. Paukner, L.R. Pacek

**Affiliations:** a Dept of Implementation Science, Wake Forest University, Winston Salem, NC, United States; b Dept of Psychiatry and Behavioral Sciences, Duke University, Durham, NC, United States; c Dept of Medicine, Duke University, Durham, NC, United States; d Dept of Biostatistics and Bioinformatics, Duke University, Durham, NC, United States; e Dept of Psychology, State University of New York at Buffalo, Buffalo NY, United States; f Dept of Biostatistics and Data Science, Wake Forest University, Winston Salem, NC, United States; g Rutgers Institute for Nicotine and Tobacco Studies, New Brunswick, NJ, United States; h Dept of Medicine, Division of General Internal Medicine, Rutgers Robert Wood Johnson Medical School, New Brunswick, NJ, United States

**Keywords:** Smoking cessation, HIV, Digital therapeutics, Pilot randomized clinical trial, Population reach

## Abstract

**Introduction:**

Tobacco use is disproportionately prevalent among people living with HIV (PWH) and is a significant contributor to morbidity and mortality in this population. Reaching communities of PWH to facilitate smoking cessation is challenging. Digital Therapeutics (DTx) can facilitate widespread implementation and adoption of smoking cessation treatments for PWH.

**Methods:**

We compared the feasibility and acceptability (primary outcomes) and preliminary efficacy (secondary outcome) of a DTx tailored to PWH – Learn to Quit-HIV (LTQ-*H*) – versus a gold standard smoking cessation DTx (QuitGuide) in a remote pilot randomized controlled trial. All participants received nicotine replacement therapy and were assessed at weeks 4, 8, and 12.

**Results:**

During a 13-month period, we remotely recruited a sample of PWH (n = 41) across the United States, with randomization leading to a higher proportion of LTQ-*H* users with high levels of cannabis use. Digital markers of DTx use indicated that compared to QuitGuide, assignment to LTQ-*H* led to significantly greater number of device interactions (3610 vs 2086; RR=93.14; 95 % CI: 14.70–590; p < 0.001), and a four-fold increase in mean interactions with active smoking cessation content (8.5 vs. 2.15; Cohen’s d=0.91; p < 0.001). At week 12, in an adjusted model, LTQ-*H* resulted in numerically greater, but not statistically significant, biochemically verified 7-day point prevalence abstinence versus QuitGuide (18.2 % vs 15.8 %; aOR=6.97, 95 % CI: 0.65–74.33).

**Conclusions:**

While participants assigned to LTQ-*H* had proportionally more features known to predict low quit rates (e.g. cannabis use), LTQ-*H* showed promising population reach, device engagement, and smoking outcomes. A hybrid effectiveness-implementation trial will evaluate this novel DTx in a larger sample of PWH.

**Implications:**

The study highlights the potential of DTx to address the high prevalence of tobacco use among people with HIV. Compared to QuitGuide (gold standard DTx developed by NCI), LTQ-*H* showed promising participant engagement and smoking cessation outcomes. These findings suggest that LTQ-*H* could be a valuable tool for smoking cessation in people with HIV, warranting further investigation in larger trials to evaluate its effectiveness and implementation feasibility.

## 1. Introduction

The prevalence of cigarette smoking among people living with HIV (PWH) is disproportionately high (47 %) (Asfar et al., 2021) compared to the United States (U.S.) general population (11.5 % in 2021) (Cornelius, 2023). Due to the development of highly effective treatments for HIV and the resulting increased longevity, smoking among PWH is now a greater cause of mortality than HIV itself (Reddy et al., 2016; Altekruse et al., 2018). An analysis of the North American AIDS Cohort Collaboration on Research and Design study (NA-ACCORD) indicated that among PWH, approximately half of smoking-related cancer and 94 % of lung cancer diagnoses could be prevented by eliminating smoking (Altekruse et al., 2018).

While a significant proportion of PWH who smoke are interested in quitting (49 %-74 %), (Pacek et al., 2017, 2014) these patients have historically had lower quit rates compared to people without HIV who smoke (Mdodo et al., 2015). Evidence suggests that traditional cessation interventions based on U.S. Clinical Practice Guidelines (USCPG) (Fiore, 2008) alone have limited effectiveness among PWH (Browning et al., 2013; Niaura et al., 2000). In a recent clinical trial, even with the use of varenicline – a first-line pharmacotherapy for smoking cessation – PWH had lower cessation rates and took longer to recover after a lapse compared to those without HIV (Ashare et al., 2021). In addition, studies show that PWH express lower interest in conventional smoking cessation modalities than non-HIV populations. In one survey, only 24.8–42.3 % of PWH who were interested in quitting smoking were interested in using various forms of nicotine replacement therapy (NRT), and less than one-third were interested in using medications like bupropion or varenicline (Pacek et al., 2017). Suboptimal cessation outcomes among PWH and relative lack of interest in conventional smoking cessation approaches (Pacek et al., 2017, 2014; Edwards et al., 2021) suggest the need for innovative, engaging, and tailored interventions for PWH (Kaufman et al., 2021; Shuter et al., 2012).

Mobile interventions range from cellphone-based interventions (e.g., text message-delivered treatments) to digital therapeutics (DTx), in which software designed to prevent or treat diseases is installed on smartphone devices (Digital Therapeutics Alliance, 2019). Mobile interventions represent a promising option to facilitate widespread implementation and adoption of smoking cessation interventions for PWH. Relative to face-to-face interventions, mobile interventions have several advantages including lower cost and greater potential to reach large numbers of PWH (Uthman et al., 2019). Additionally, mobile interventions can be advantageous from an adoption standpoint, as many HIV providers report limited training regarding smoking cessation interventions and limited confidence in their ability to impact smoking behavior (Crothers et al., 2007). Prior work indicates the feasibility and acceptability of smoking cessation interventions delivered via cellphone, (Vidrine et al., 2006, 2012; Aigner et al., 2017) text message, (Tseng et al., 2017) and smartphone (Shuter et al., 2018; McClure et al., 2024) among PWH. Moreover, a meta-analysis concluded that mobile interventions may be more efficacious than face-to-face interventions for smoking cessation among PWH in the short term, but that new approaches may be needed to sustain long-term effects (Uthman et al., 2019). In addition to the promising evidence offered by cessation interventions delivered via this modality, it is important to consider the substantial data supporting tailored behavior change interventions, (Noar et al., 2007; Lustria et al., 2013) suggesting the need to develop mobile smoking cessation interventions that address the unique needs of PWH.

Co-morbid psychological disorders, HIV-related, and cognitive deficits (Nanni et al., 2015; Babel et al., 2021; Deng et al., 2021). represent potential barriers to cessation among PWH. Therefore, we conducted a user-centered design study (manuscript in preparation) to adapt and tailor an existing evidence-based DTx for smoking cessation called Learn to Quit (Vilardaga et al., 2018) to address these three specific factors. The resulting digital intervention, Learn to Quit-HIV (LTQ-*H*), combines USCPG (Fiore, 2008) and Acceptance and Commitment Therapy (ACT) (Hayes et al., 2011). ACT is a behavioral intervention originally designed to treat psychological disorders that has been shown effective for smoking cessation in previous trials (Gifford et al., 2011; Bricker et al., 2020, 2018). Adapting ACT to help PWH who smoke offers several advantages and opportunities for this population. Negative affect, stress, and mental health symptoms are established predictors of smoking relapse, (Kinnunen et al., 1996; Lerman et al., 1996; Niaura et al., 1999) all of which are common experiences in PWH (Bing et al., 2001; Miles et al., 2019). ACT has theory-based components (i.e., psychological flexibility) that predict cessation (Vilardaga et al., 2014; Heffner et al., 2015; Browne et al., 2021; Bricker et al., 2021) and works by targeting two core skills: (a) acceptance (vs control) of the cognitive, emotional, and physical sensations associated with urges to smoke, and (b) commitment to engage in behaviors that are consistent with stated goals and values (Vilardaga et al., 2014). These core skills of ACT predict both proximal (Vilardaga et al., 2014) and distal cessation outcomes (Heffner et al., 2015). LTQ-*H* modified six of the fourteen core modules of LTQ to include HIV-relevant content (e.g., psychoeducation about the health impact of HIV and smoking, HIV stigma), added weekly and monthly summary screens, and a new library of notifications.

The present paper reports the results of a pilot randomized controlled trial (i.e., Quitting Matters Pilot; NCT04609514) to evaluate the feasibility, acceptability, and smoking outcomes of LTQ-*H* compared to an active control app (i.e., QuitGuide). The Quitting Matters Pilot also evaluated the implementation and potential reach of offering smoking cessation treatment in this modality by recruiting PWH in a fully remote trial. We hypothesized that conducting a remote trial testing a DTx among PWH would be feasible and acceptable, and that LTQ-*H* would result in higher levels of usability and DTx engagement compared to QuitGuide. Secondary outcomes included number of cigarettes smoked per day, quit attempts, and biochemically verified smoking abstinence. No specific benchmarks were originally proposed for these hypotheses.

## 2. Methods

### 2.1. Design

This study was a 12-week parallel 2-arm randomized controlled trial with virtual follow-up assessments at 4-, 8-, and 12-weeks post-randomization comparing LTQ-*H* to QuitGuide, a digital intervention matched to LTQ-*H* in terms of length and means of delivery to control for the effects of time and attention (NCT04609514).

### 2.2. Participants

Eligibility criteria included: (1) self-reported HIV-positive status, (2) currently under the care of an HIV provider, (3) self-reported smoking of ≥ 5 cigarettes per day during the past 30 days, (4) ≥ 18 years of age, (5) current interest in quitting smoking, (6) current ownership of an Apple or Android smartphone. Exclusion criteria were: (1) concerns about participant safety by study physician, participant physician, or study Principal Investigators, or inability to provide informed consent, (2) contraindication or prior side effects associated with nicotine patch, (3) current use of NRT or other smoking cessation medication, (4) being pregnant, planning to become pregnant, nursing, or becoming pregnant during the study, and (5) untreated and unstable diagnosis of substance use disorder within the last 30 days.

All participants signed a release of information form with the contact information of their current physician or clinical provider to address potential concerns about eligibility prior to randomization and/or clinical safety during participation in the trial.

### 2.3. Interventions

#### 2.3.1. LTQ-H

LTQ-*H* is a DTx that can be installed on both Android and Apple iOS operating systems. LTQ-*H* consists of 324 screens of content divided into: (a) HIV-tailored smoking cessation ACT skills, (b) education about tobacco dependence and treatment, and (c) psychoeducation about NRT and adherence. Content is gradually presented across 28 modules (14 education and 14 skills) that can be completed in no less than 14 days. The modules encourage the learning and practice of three processes of change (i.e., awareness of urges to smoke, openness to experiencing urges, commitment to specific values for quitting). The DTx also includes a self-tracking feature of mood, NRT use, cravings to smoke, acceptance of cravings, and cigarettes smoked daily (CPD). These assessments are not incentivized or necessary to complete the other DTx content or modules. A more detailed description about the design and adaptation of LTQ-*H* is currently under review.

#### 2.3.2. QuitGuide

QuitGuide is a DTx based on USCPG for smoking cessation developed for the general population by the Smokefree.gov initiative of the Division of Cancer Control and Population Sciences of the National Cancer Institute (National Cancer Institute, 2022). The intervention includes the following components: (a) psychoeducation about the impact of smoking on health, (b) setting up a quit date and a quit plan, (c) selecting reasons for quitting, (d) tracking of mood, triggers, smoking habits, and (e) tips for quitting. It also contains a journaling feature and the option to opt-in to location- or time-based notifications. More information about QuitGuide’s rationale and content can be found at www.smokefree.gov.

#### 2.3.3. Nicotine replacement therapy

All participants received an 8-week course of transdermal nicotine patches starting at 21 mg/24 h, and tapered following recommendations contained in the USCPG (Fiore, 2008).

### 2.4. Procedures

Participants were recruited through patient referrals from other HIV research studies, infectious disease clinics, and the electronic health record at Duke University School of Medicine, as well as through community clinics in the Durham, N.C. area. Due to national guidelines in response to the COVID-19 pandemic, our trial included procedures to conduct a fully remote pilot trial with expanded recruitment nationwide through online advertisements (e.g. Facebook, Craigslist).

Interested participants were instructed to contact the lab by phone or email to learn more about the study. A phone screening was conducted to assess initial eligibility, followed by a remote video screening visit (week - 1) during which informed e-consent was obtained and additional eligibility for the study were evaluated. Final eligibility was confirmed by the study physician and/or principal investigators. Eligible participants were randomized 1:1 to either LTQ-*H* or QuitGuide (week 0). This two-step procedure of participant enrollment was designed to allow verification of inclusion criteria and mitigate participant deception (e.g., online professional participants). Research staff guided the randomized participants to install the assigned DTx on their smartphone device. Participants were given instructions to set a quit date within their assigned DTx as well as complete modules and track cigarette use. To protect participants’s privacy, LTQ-*H*’s notifications excluded any HIV-related messaging as a safeguard to prevent potential disclosure of HIV status. Participant onboarding for each DTx was limited to providing a download link and installation code, consistent with the standalone nature of this digital intervention and to maximize scalability and implementation potential.

Participants continued to receive HIV care as provided by their usual healthcare provider. Virtual visits occurred at 4-, 8-, and 12-weeks post-randomization. Among participants who self-reported abstinence at 12-weeks post randomization, study staff conducted remote (i.e., via Zoom video call) biochemical verification of smoking abstinence using Covita’s iCOquit Smokerlyzers (Bedfont Scientific Ltd). Participants received $130 compensation for participation in all study activities. All study procedures were approved by the Institutional Review Board at Duke University School of Medicine.

### 2.5. Measures

#### 2.5.1. Baseline characteristics

##### Tobacco Use Behavior.

At baseline, we collected self-reported years of smoking, nicotine dependence, (Heatherton et al., 1991) CPD, frequency of e-cigarettes use, and past 30-day use of other tobacco products.

##### HIV-Related Characteristics, Social Determinants of Health, and Mental Health Factors.

We collected information regarding the duration (in years) of HIV diagnosis, and self-reported current CD4 (T-Cell) counts. Other HIV-related measures included internalized HIV stigma using a subscale of the HIV Stigma Mechanisms Scale, (Earnshaw et al., 2013) with scores ranging from 6 to 30 for which high scores indicate greater stigma. Self-perceived discrimination was measured with the Everyday Discrimination Scale, (Krieger et al., 2005) with responses ranging from 5 through 30 and with lower scores indicating higher levels of discrimination.

At baseline we administered the Depression Anxiety Stress Scale (DASS-21) (Lovibond and Lovibond, 1995) and the Brief Symptom Inventory (BSI) (Derogatis, 1992). DASS-21 is a self-report measure of symptoms of depression, anxiety, and stress. Scores for each subscale are categorized into normal, mild, moderate, severe and extremely severe categories. The Global Severity Index is a subscale of the BSI that captures a wider range of psychiatric symptoms, with scores ranging from 0 to 4 and with higher scores indicating more psychiatric severity.

##### Digital Divide and Familiarity with Technology.

All participants completed a questionnaire internally developed by our team in prior research assessing technology access, familiarity, and use. This survey asked about previous experiences with smartphones, internet availability, range of activities for internet use, and concerns about the use of technology as a health resource or tool.

##### Substance Use.

Substance use behaviors were examined via self-report surveys including the Drug Abuse Screening Test-10 (DAST-10) and the Alcohol Use Disorders Identification Test (AUDIT), (Skinner, 1982) two widely used and reliable measures with clinical cut-offs of 3 and 8 respectively (Saunders et al., 1993; Yudko et al., 2007). Self-reported daily cannabis use (yes/no), number of days of cannabis use within the last 30 days, and mixing cannabis and tobacco products (yes/no) were collected at baseline with three single items.

#### 2.5.2. Primary outcomes

##### 2.5.2.1. Recruitment rate, participant attrition, and population reach.

Recruitment and participant retention rates were monitored and recorded throughout the study duration. The demographic characteristics of participants and their state of residence were collected at baseline. The pilot trial also tracked the number and geographic location of HIV clinics reached for the purpose of trial advertisement and participant recruitment.

##### 2.5.2.2. Digital markers of DTx use.

Based on user interactions defined in prior work (see Supplementary Table 1 (Vilardaga et al., 2019)), we used Google Analytics to collect digital markers of LTQ-*H* use from device metadata. For QuitGuide, metadata was obtained directly from the NCI’s smokefree.gov program via a Data Transfer Agreement. Intensive longitudinal data from each device was grouped and organized to evaluate different metrics of device engagement as shown in Table 2. Specifically, interactions with active smoking cessation content for LTQ-*H* were extracted from unique counts of any smoking cessation module completed (i.e., 28 modules), which included ACT content in combination with USCPG. For QuitGuide, we extracted unique counts of any new engagement with the following USCPG content: setting up quit date, setting up a quit plan, selecting reasons for quitting, reviewing ‘How to Quit’ guide, or smoking trigger-based tips and feedback.

##### 2.5.2.3. DTx usability.

DTx’s usability was measured with the Systems Usability Scale (SUS), a 10-item questionnaire with a 5-point Likert scale from strongly agree to strongly disagree and with possible total scores ranging from 0 to 100 (Brooke, 1996). A mean score of 68 is considered a useful benchmark for assessing usability, where 50 % of devices fall below the benchmark of 68 (Hyzy et al., 2022).

#### 2.5.3. Secondary outcomes

##### 2.5.3.1. Smoking outcomes.

Secondary outcomes for this pilot trial included *quit attempts* defined as self-reported no smoking at all for 24 h from baseline to Week 12, *change in CPD* from baseline to Week 12, and *biochemically verified 7-day point prevalence abstinence* (i.e., not smoking even a puff of a cigarette within the last 7 days, and less than 5 parts per million of carbon monoxide on a breath test at week 12) (Perkins et al., 2013; Cropsey et al., 2014; Hughes et al., 2003). *Adherence to NRT* was measured daily with a brief interviewer-administered questionnaire at Weeks 4, 8, and 12 to assess the overall proportion of dispensed NRT used as directed, measured as a continuous variable. Participant safety was assessed by monitoring all adverse events during the 12-week period and in close collaboration with their medical providers.

### 2.6. Data analytic strategy

Descriptive statistics were used to report recruitment and participant retention rates. Trajectories of DTx engagement were plotted to examine ongoing interactions with each device. Acceptability outcomes were evaluated descriptively with summary scores of the SUS. Group differences in secondary outcomes were assessed via *t*-tests, logistic regression, or linear regression, as appropriate. To appropriately account for negatively skewed count distributions and the nested nature of the intensive longitudinal count data we conducted zero inflated negative binomial multilevel models that compared the trajectories of DTx engagement across treatment arms. T-tests were used to evaluate utilization of active smoking cessation content within each DTx. Logistic regression was used to evaluate the differences in biochemically verified 7-day point prevalence abstinence using the full intent to treat sample (missing = smoking). Logistic regression models were adjusted for baseline variables with an observed imbalance across arms (i.e., baseline levels of psychological stress, daily cannabis use, mixed cannabis and tobacco use, and number of cigarettes per day). T-tests and linear regression were used to evaluate the differences in change in cigarettes per day, quit attempts, adherence to NRT and participant safety. No alpha corrections were conducted for this analysis given the pilot nature of this study (Leon et al., 2011). All statistical analyses were conducted with R version 4.4.2.

## 3. Results

### 3.1. Pilot trial recruitment and retention

**Fig. 1.**
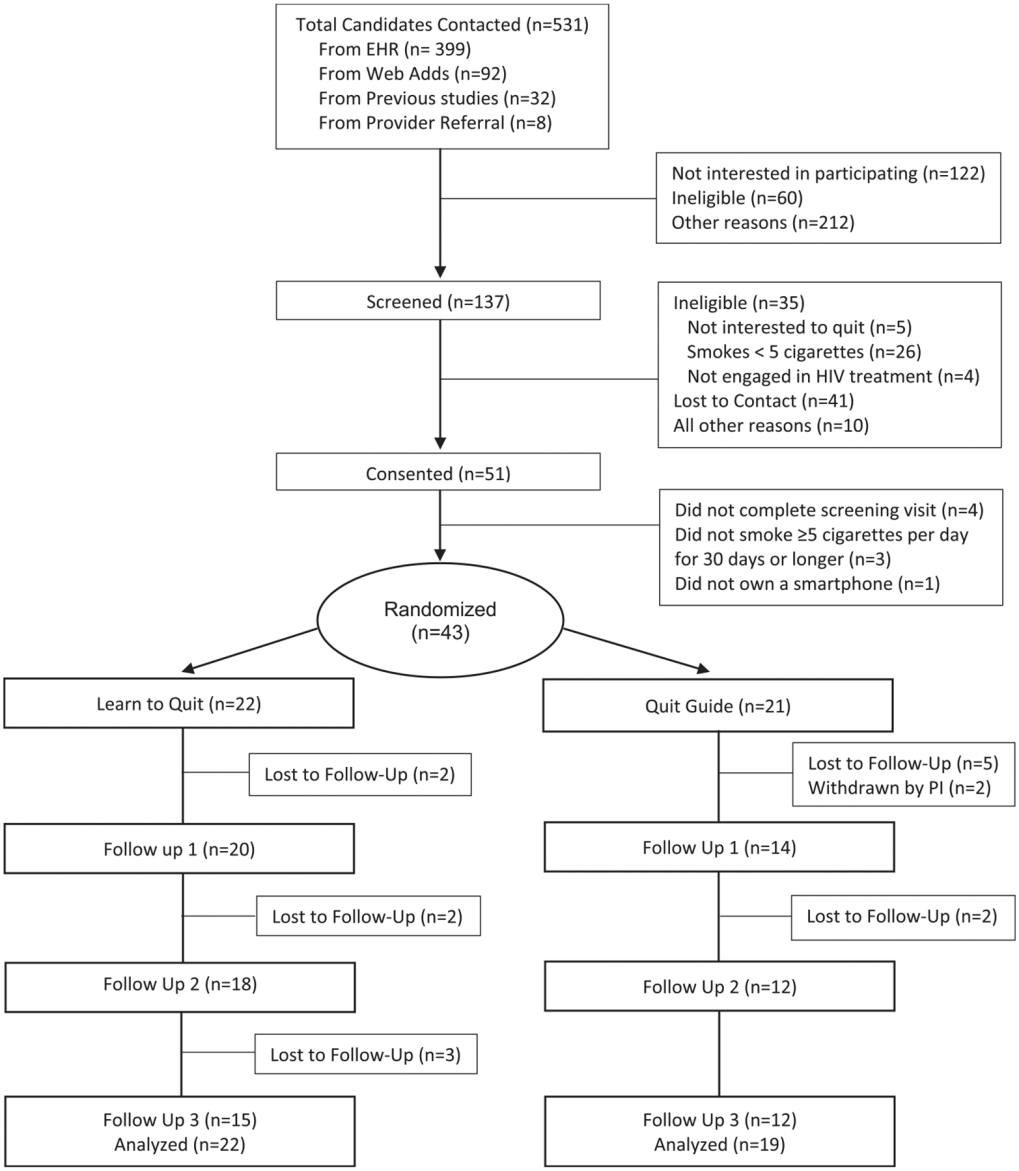
Consort chart for Quitting Matters pilot trial (n=43).

Recruitment of participants took place between February 22, 2021 and March 2, 2022 at a rate of 3.5 participants per month. The last participant completed the study on June 3, 2022. A total of 531 potentially eligible individuals (i.e., self-reported smoking and HIV) were contacted for an initial phone screening. Among the 51 PWH who completed informed consent procedures, 8 were not randomized due to failing screening procedures or not completing the screening visit. Forty-three participants were randomized to a treatment condition, and 2 were withdrawn by the PI due to new information obtained after randomization indicating that these participants did not meet the eligibility criteria (see CONSORT chart in Fig. 1). The pilot trial had a retention rate of 78 %, defined as all randomized participants who responded to our last follow-up survey.

**Fig. 2.**
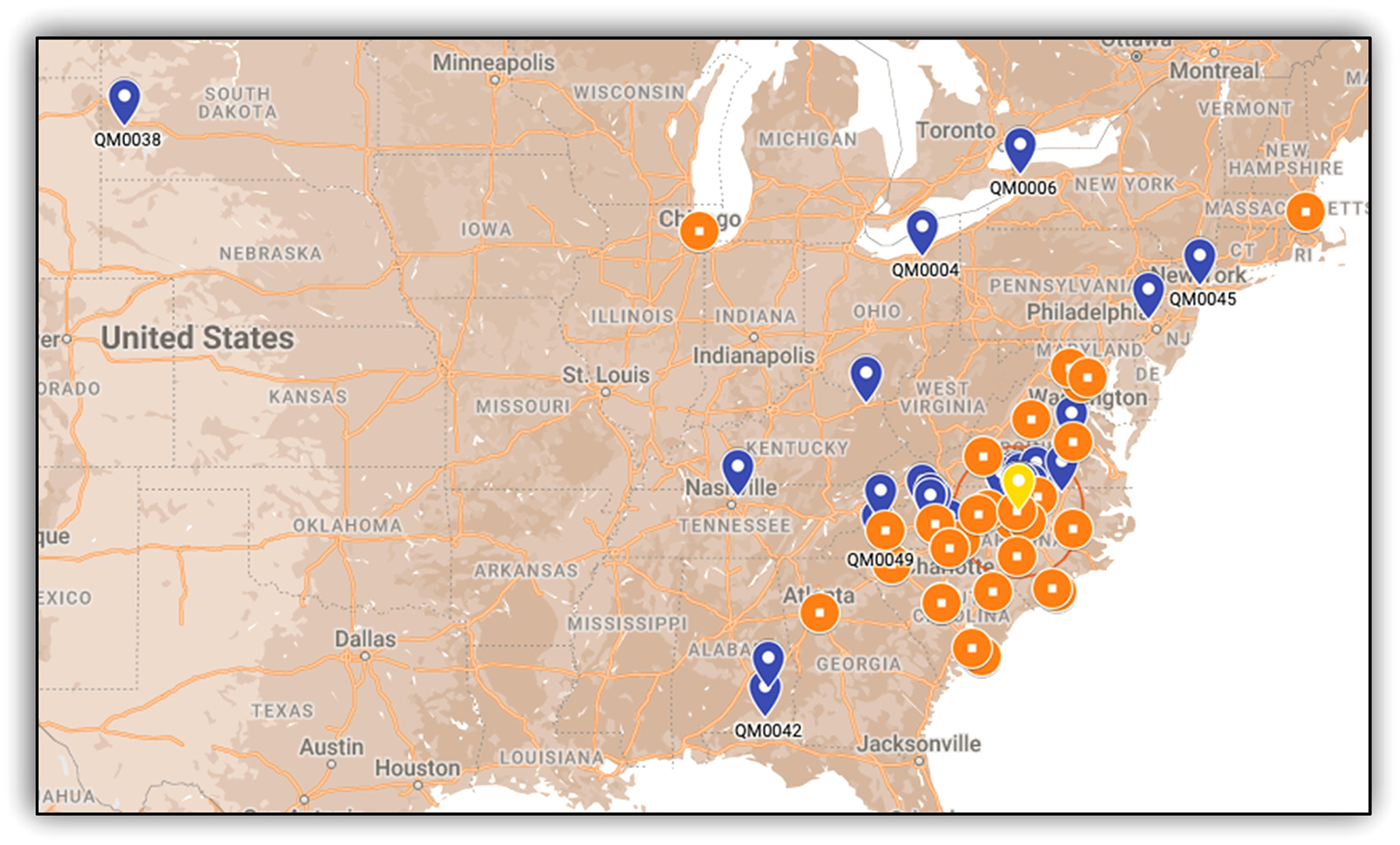
Geographical distribution of county of residence for participants with HIV and HIV clinics in the Quitting Matters Pilot Trial. Note. Blue (or dark) marks: study participants; Orange (or light) marks: HIV clinics; Yellow mark: clinical trial site.

To obtain preliminary data about the potential reach of DTx for smoking cessation among PWH populations, we contacted 31 HIV clinics and two smoking cessation centers across the U.S. (i.e., orange dots in Fig. 2) to introduce our study and provide recruitment materials. Sixteen clinic administrators responded to our initial email, and among those, ten clinics expressed interest in a 10-minute introductory session to their team to discuss the study. Finally, we presented our study to the clinical and administrative leadership of nine clinics; all agreed to post our flyers at their clinic locations and/or refer their patients to our study line. In sum, 30 % of the clinics we contacted formally or informally advertised the study to their patient pool.

### 3.2. Participant characteristics

The final sample (n = 41) was racially diverse with approximately equal proportions of White and Black participants (Table 1). More than 75 % of participants reported having high school or fewer years of education, and having lived with a diagnosis of HIV for almost two decades. Randomization to treatment arms rendered comparable participant characteristics across basic demographics, HIV medical history, drug and alcohol use, HIV stigma, experiences of discrimination, and familiarity with technology. However, relative to QuitGuide, LTQ-*H* arm participants were more likely to have above normal levels of psychological stress (36 % vs 11 %), higher prevalence of any cannabis use in the past 12 months (54 % vs 10 %), more days of cannabis use in the past 30 days (mean 16 days vs 8 days), higher percentage of self-reported mixing cannabis and combustible tobacco products (18 % vs 5 %), more baseline cigarettes use per day (mean 16.05 vs 12.22), higher prevalence of e-cigarettes use (9 % vs 0 %), and higher prevalence of other tobacco products (27 % vs 5 %). Scores on the DAST-10 and the AUDIT were also similar across arms. Across arms, participants reported low levels of perceived discrimination and internalized HIV stigma (see Table 1).

### 3.3. Digital markers of device engagement

**Fig. 3.**
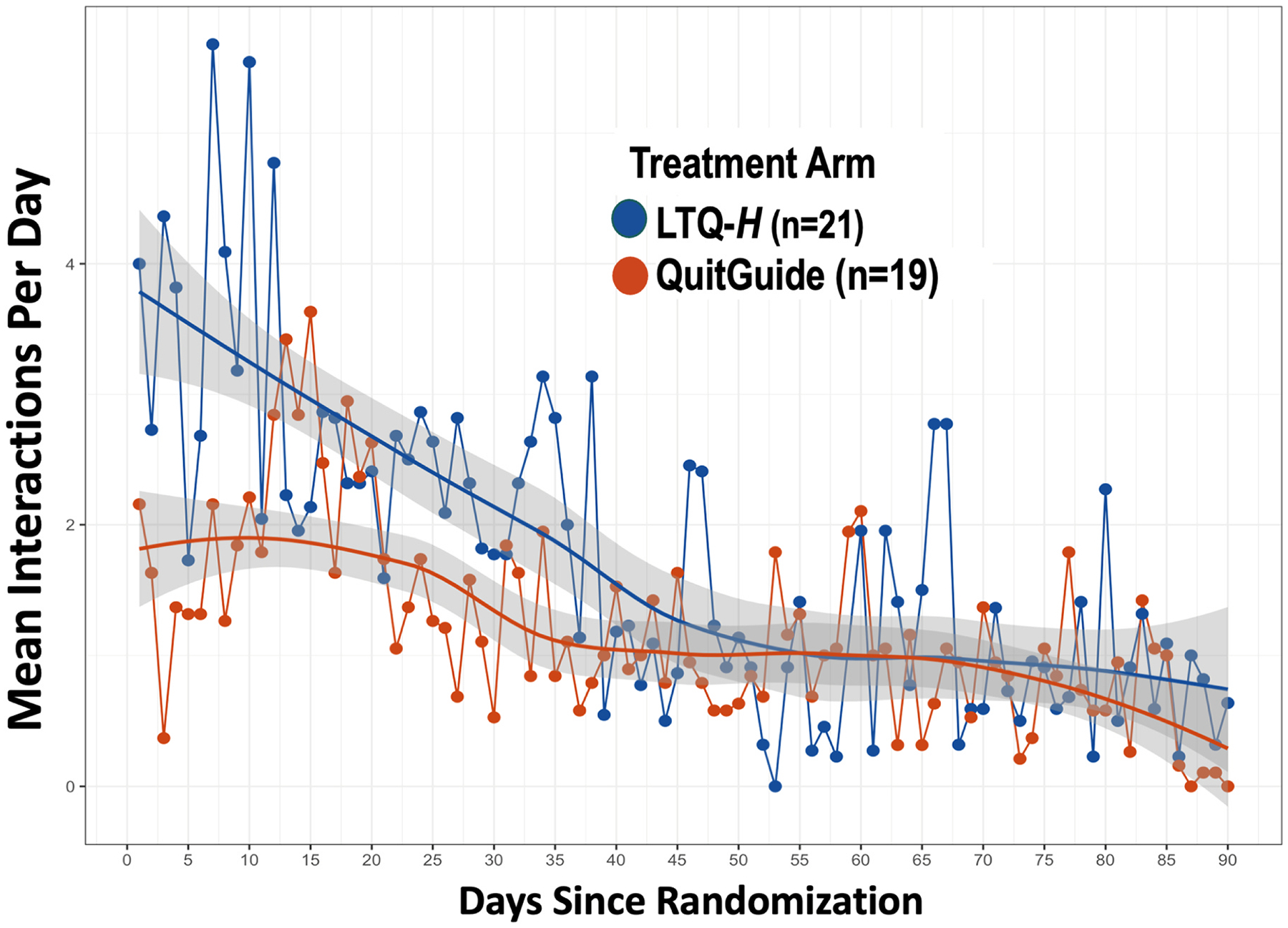
Mean DTx interactions per day by study arm with non-parametric locally estimated scatterplot smoothing (loess) lines and corresponding standard error shaded areas (n = 41). Note. Seven participants in the LTQ-H did not generate digital markers data due to mechanical error caused by a software upgrade.

Seven participants in the LTQ-*H* arm (33 %) generated only partial digital markers due to mechanical error caused by a software upgrade. When partial digital markers were included, participants in the LTQ-*H* arm had 1524 more interactions with LTQ-*H* than QuitGuide (3610 vs 2086), with an average of interactions per day of 1.82 (SD=2.11) compared to 1.21 (SD=4.28) for QuitGuide. During the first 30 days of DTx use, the average was of 2.89 (SD=2.98) for LTQ-*H* versus 1.82 (SD=6.48) for QuitGuide. A zero-inflated negative binomial multilevel count model indicated that these differences were statistically significant. The LTQ-*H* arm also had a significant and larger effect than QuitGuide on the count of days of device use using the same statistical model. LTQ-*H* users completed close to four times as much active smoking cessation content as QuitGuide users. Only 5 participants in the QuitGuide arm (26 %) versus 22 (100 %) participants in the LTQ-*H* engaged with active smoking cessation content. See Table 2 for all results and Fig. 3 for raw interaction data.

### 3.4. Device usability

LTQ-*H* and QuitGuide had scores on the SUS above the standard cut-off (i.e., 68; See Table 2) that were not statistically different between arms.

### 3.5. Smoking reductions, quit attempts, and smoking abstinence

There were no differences in absolute reductions in CPD in LTQ-*H* vs QuitGuide. QuitGuide participants, compared to LTQ-*H* participants, made a larger but not significantly different mean number of quit attempts. Biochemically verified 7-day point prevalence abstinence at week 12 for the intent-to-treat sample was 18.2 % (4/22) for LTQ-*H* vs 15.8 % (3/19) for QuitGuide. Our statistical model adjusted for baseline levels of cigarettes smoked per day, days of cannabis use in the past 30 days, mixed cannabis and combustible tobacco use, and psychological stress. In completer’s analysis (n = 32) adjusted for the same variables, biochemically verified 7-day PPA was 23.5 % for LTQ-*H* versus 20.0 % for QuitGuide (see Table 2 for all statistical values).

### 3.6. Nicotine replacement patch adherence and participant’s safety

Adherence to the nicotine patch was similar across treatment arms (31.0 % for LTQ-*H* vs 31.6 % for QuitGuide) and not statistically different. Similarly, no serious adverse events were reported during the study, and no statistical differences in number of adverse events were identified by study arm (see Table 2).

## 4. Discussion

The Quitting Matters Pilot demonstrated that it is feasible to reach a demographically and geographically diverse sample of PWH for the purpose of remotely delivering smoking cessation interventions for this population using a mobile digital device (e.g., DTx). Furthermore, it demonstrates that DTx can lead to high levels of treatment engagement and utilization with these technologies in this population. Our remote trial was able to reach PWH from 10 US states, as well as HIV clinics across the Eastern US and engage with a sizeable portion of contacted clinics. Relative to QuitGuide, a standard of care DTx for smoking cessation developed by the National Cancer Institute that has shown efficacy in prior trials, (Bricker et al., 2020) our tailored DTx for PWH (LTQ-*H*) achieved comparable levels of device usability, but significantly higher rates of days of use, total amount of device engagement, and targeted interactions with active smoking cessation content. These levels of engagement were assessed during a 90-day period using state-of-the-art statistical approaches for intensive longitudinal data. This finding is important, since in a previous DTx study, we demonstrated that device engagement with LTQ, and not QuitGuide, causally mediated smoking outcomes at trial endpoint (Browne et al., 2021). Further, more than 75 % of PWH recruited in this study had high school or fewer years of education which demonstrates that the LTQ-*H* device engaged a population traditionally underrepresented in technology-based interventions (Mackert et al., 2016; Kontos et al., 2014). Individuals with lower educational attainment often face barriers to accessing and benefiting from digital health tools (Berkman et al., 2011). Therefore, successfully engaging this group suggests that the LTQ-*H* device is both usable and engaging across diverse educational backgrounds, ensuring that digital innovations do not exacerbate existing health disparities (Latulippe et al., 2017).

While the Quitting Matters Pilot trial was not powered to detect statistical differences between arms in smoking outcomes, LTQ-*H* had descriptively higher rates of biochemically confirmed smoking abstinence, and larger smoking reductions from baseline to trial endpoint. It is important to note that our randomization procedure failed to equally distribute participants across baseline features known from previous research to predict low quit rates, such as cannabis use, mental health symptoms, and smoking intensity (Ni et al., 2020; Streck et al., 2020; Rogers et al., 2020). More specifically, participants randomized to LTQ-*H* used cannabis on twice the number of days in the prior thirty days compared to participants randomized to QuitGuide, had three times greater levels of psychological stress than QuitGuide participants, and smoked more CPD at baseline. Moreover, biochemical verification of one participant assigned to the LTQ-*H* arm failed likely due to self-reported combustible cannabis use prior to the verification test, which is consistent with the fact that 18 % of LTQ-*H* participants (vs 5 % for QuitGuide) reported at baseline mixing cannabis and combustible tobacco products.

The results of this pilot trial suggested that conducting a remote clinical trial of digital interventions for smoking cessation in this population was acceptable but may require a more robust recruitment strategy (e.g., higher incentives and/or clinic involvement). Further, the use of biochemical verification improved the accuracy of our outcomes. However, combustible cannabis use limits this approach to biochemical verification, which may have reduced our overall quit rate for the LTQ-*H* arm. The Quitting Matters pilot suggests that future studies should use a combination of CO and cotinine samples to minimize false positives, which has been tested in remote studies (Raiff et al., 2022; Kong et al., 2017) and is consistent with recent guidelines (Benowitz et al., 2020).

The largest study examining the use of a digital intervention for smoking cessation in this patient population found quit rates of 14.9 % (Shuter et al., 2022). Other pilot studies like the one reported here found quit rates ranging from 15 % to 40 %, (Schnall et al., 2022; Poudel et al., 2023; Bui et al., 2022) yet those studies used different endpoints (e.g., 2-month follow-up), (Bui et al., 2022) international populations (e.g., Cambodia) (Poudel et al., 2023; Bui et al., 2022) or reported different primary smoking outcomes (e.g., smoking reductions) (McClure et al., 2024). Together with the fact that LTQ-*H* demonstrated high levels of device engagement during the three months trial period, these smoking outcomes suggest that LTQ-*H* holds great promise for the remote delivery of smoking cessation interventions among PWH.

From an implementation science standpoint, the levels of HIV clinic outreach of the Quitting Matters Pilot are particularly significant in the context of the COVID-19 pandemic. One of the clinic directors stated that, “*My professional life has been completely overtaken by COVID*.” We believe this statement represented the experience of many infectious disease clinics in the U.S. at the time we recruited for this pilot study. In that context, the proportion of clinics that expressed interest in our study provides preliminary support for the implementation of LTQ-*H* in HIV clinics throughout the country.

Our pilot trial had several limitations. First, our recruitment rates were slightly low relative to the total number of candidates that were initially contacted, which is typical of trials recruiting patients for cancer treatment and low prevalence diseases or conditions such as HIV (Lara et al., 2001; Haider, 2022). However, these low recruitment rates call for a more robust national recruitment strategy in this patient population. Our two-step procedure of participant enrollment and verification (i.e., one screening visit followed by a randomization visit) may have reduced our final enrollment rates, but this procedure also mitigated participant deception, a common occurrence in online clinical trials (Heffner et al., 2021). Low recruitment rates can also be expected from pilot studies with a moderate budget for personnel and advertisement such as this pilot. Overall, one year of recruitment generated a sample size that was fully adequate for the goals of a pilot trial (Eldridge et al., 2016a). Second, our biochemical verification relied solely on expired breath CO, which has inherent limitations (e.g., it cannot confirm abstinence beyond 3–5 days) and may have been inappropriate for a population with high levels of combustible cannabis use. Rogers et al., (2020); Rice et al., (2022). Data from this pilot study also suggests a more adaptable biomarker collection procedure (both CO and cotinine) to avoid sample contamination from e-cigarettes, or NRT use, as conducted in prior remote trials (Vilardaga et al., 2019; Brooke, 1996). The use of anatabine/anabasine to capture combustible tobacco products could also be considered. A more adaptable biomarker collection approach is consistent with most recent SRNT taskforce guidelines (Benowitz et al., 2020). Third, notable imbalances between treatment arms in patient characteristics known to predict low quit smoking rates may have biased our study in favor of the QuitGuide app. Larger trials may consider stratified randomization to account for the strong influence of these patient characteristics. Fourth, both LTQ-*H* and QuitGuide directly encouraged the use of NRT. However, adherence to the NRT patch was low (<32 %), and did not differ between arms. These low adherence rates were consistent with prior literature, (Pacek et al., 2018) and in this study could also be explained by the lack of in-person contact with a provider (low-touch medical oversight), a clinical tradeoff between more intensive treatment modalities and remote digital trials that ultimately favors scalability and high reach. Finally, while the study was able to collect a significant number of device interactions with LTQ-*H* and QuitGuide, an unexpected software upgrade led to a loss of 32 % of device interactions in the LTQ-*H* arm. However, the statistical approach we chose to analyze this data provided robust parameter estimates to account for individual and group variability in intensive longitudinal datasets and ample statistical power to evaluate our device engagement hypotheses.

In conclusion, the Quitting Matters Pilot trial demonstrated that LTQ-H, a novel digital intervention, has the potential to engage a racially and demographically diverse population of PWH with low literacy, may achieve higher user engagement and acceptability than a DTx designed for the general population, and shows promising smoking cessation outcomes. This pilot also established the feasibility of conducting a fully remote trial with biochemical verification in this population and fostered community partnerships with HIV clinics. Building on these findings, a fully powered, nationwide remote hybrid Type 1 effectiveness-implementation trial is currently underway to evaluate the effectiveness and implementation potential of LTQ-H among a larger sample of PWH across the U.S. (NCT06883097).

## Figures and Tables

**Table 1:** Baseline characteristics of pilot trial participants (N = 41).^1^.

Variables	LTQ-H	QuitGuide
**Demographics**	n = 22	n = 19
Age	48.5 (11.12)	52.84 (9.45)
Female	22.72 %	36.84 %
White	50.00 %	47.00 %
Black	45.00 %	47.38 %
Other Race	0.00 %	5.26 %
Hispanic American	4.54 %	5.26 %
Education (≤ High School)	77.27 %	89.47 %
Unemployed or Disability Status	36.36 %	31.58 %
Household Income (<$35,000)	45.50 %	42.11 %
**HIV Characteristics**		
Years Diagnosed with HIV-Positive	18.65 (9.58)	19.18 (8.31)
CD4/T-cell count^2^	893.40 (447.55)	775.33 (410.81)
**Mental Health Symptoms**		
% Above Normal Depressive Symptoms^3^	0.00 %	0.00 %
% Above Normal Anxiety Symptoms^3^	9.09 %	10.52 %
% Above Normal Psychological Stress^3^	36.36 %	10.52 %
Overall Psychiatric Functioning^4^	0.24 (0.31)	0.20 (0.31)
**Substance Use**		
Drug Abuse Screening Test (DAST)	3.00 (1.15)	3.00 (2.83)
Alcohol Use Disorders Test (AUDIT)	3.73 (3.01)	2.00 (1.00)
Any Cannabis Use Past 12 Months	54.54 %	10.53 %
Days of Cannabis Use Past 30 days	16.83 (12.01)	8.00 (9.90)
Reported Mixing Cannabis with Tobacco at Baseline	18 %	5%
**Psychosocial Factors**		
Everyday Discrimination Scale	25.14 (7.92)	29.00 (1.89)
Internalized HIV Stigma	10.22 (5.66)	8.42 (3.84)
**Familiarity with Technology**		
Internet Availability at Home	100 %	100 %
Wide Range Use of Technology (3 Activities)	86.37 %	78.95 %
Expressed Concern with Technology as Health Resource	0 %	0 %
**Tobacco Use Behavior**		
Cigarettes Per Day	16.05 (6.79)	12.22 (5.42)
Years Smoking Cigarettes	26.91 (13.31)	27.28 (11.36)
Nicotine Dependence^5^	4.73 (1.62)	4.00 (1.80)
E-Cigarettes	9.09 %	0.00 %
Any Other Tobacco Products	27.27 %	5.26 %

Note.

1: Baseline characteristics are reported based on Mean (M) and Standard Deviations (SD) unless otherwise specified.

2: Self-reported CD4/T-cell counts;

3: Percentages for each category were extracted from cut-offs of the Depression, Anxiety and Stress Scales-21.

4: Global Severity Index of the Brief Symptom Inventory;

5: Measured with the Fagerström Test for Nicotine Dependence.

**Table 2:** Population reach, feasibility, acceptability and smoking outcomes of the Quitting Matters pilot (N = 41)^1^.

Pilot Trial Feasibility and Reach	All Arms			
Accrual duration and rate	13 months, 3.5 per month			
Participant retention	78 %			
Reach of remote pilot trial	10 US states			
HIV clinics’ reach	30 % response rate			
Acceptability of Digital Therapeutic	LTQ-H	QuitGuide	Effect^2^	p-value
Average interactions all 90 days	164 (189)	109 (385)	RR= 93.14 (14.70, 590)^3^	p < 0.001*
Average days of use	14.40 (16.26)	9.42 (21.18)	RR= 7.84 (2.12, 29.04)^3^	p = 0.002*
Mean interactions with active content^4^	8.50 (5.20)	2.15 (5.89)	d= 0.91 (0.45, 1.37)^5^	p < 0.001*
Mean System Usability Scale^6^	73.00 (16.25)	77.96 (15.63)	d= −0.31 (−0.99, 0.37)^7^	p = 0.36
**Smoking Behavior**				
Number of Quit Attempts	1.857 (1.68)	2.50 (1.38)	d= −0.41 (−1.65, 0.82)^5^	p = 0.46
Absolute Reductions in CPD^8,9^	11.06 (4.91)	9.27 (5.92)	d= 0.27 (−0.52, 1.08)^5^	p = 0.50
Adj 7-day Bioverified PPA Wk12^11,12^	18.2 % (4/22)	15.8 % (3/19)	aOR= 6.968 (0.65, 74.33)	p = 0.11
Adj 7-day Bioverified PPA, Wk 12, Completers.^11,13^	23.5 % (4/17)	20 % (3/15)	aOR= 6.170 (0.57, 66.98)	p = 0.13
**Other Clinical Outcomes**				
Mean Adverse Events	2 (1.09)	1.5 (0.54)	d = 0.57 (−0.73, 1,89)^5^	p = 0.58
Adherence to Nicotine Patches^11,14^	31.82 %	31.6 %	OR= 1.01 (0.270, 3782)	p = 0.99

1: All values in parenthesis represent Standard Deviations unless otherwise specified.

2: Effects are reported as Risk Ratios (RR), Odd Ratios (OR), and Cohen’s d [small effect (d=0.2); medium effect (d=0.5), large effect (d=0.8)], and corresponding 95 % confidence intervals. Significant tests are noted with an asterisk.

3: Zero Inflated Negative Binomial multilevel model.

4: For LTQ-H, the mean is based on counts of any new smoking cessation learning module completed. For QuitGuide, the mean is based on counts of any new engagement with smoking cessation content consistent with U.S. Clinical Practice Guidelines (i.e., setting up quit date, setting up a quit plan, selecting reasons for quitting, reviewing ‘How to Quit’ guide, or triggered-based tips and feedback).

5: two sample *t*-test.

6: Standard cut-off: > 68.

7: Linear regression model.

8: CPD=cigarettes per day.

9: Absolute difference between reductions in CPD between arms.

10: Relative reductions in CPD between arms.

11: Logistic regression model with estimates adjusted to baseline levels of stress, daily marijuana use, mixed cannabis and combustible cannabis use, and number of cigarettes per day; Adj: Adjusted; Wk: week

12: Intent to treat sample.

13: Completers: Completer’s analysis (n = 32).

14: Percent was calculated by dividing self-reported days of patch use from baseline to week 8 by 60 days (8 weeks) relative to reaching an 80 % adherence benchmark.

## Glossary

HIV: Human Immunodeficiency Virus
PWH: People with HIV
DTx: Digital Therapeutics
LTQ-H: Learn to Quit HIV
ACT: Acceptance and Commitment Therapy
USCPG: United States Clinical Practice Guidelines
EHR: Electronic Health Record
NRT: Nicotine Replacement Therapy
CO: Carbon Monoxide
CPD: Cigarettes per Day
PPA: Point Prevalence Abstinence
N: total sample size
CI: Confidence Interval
p: probability value
d: difference
